# Catechin Composition, Phenolic Content, and Antioxidant Properties of Commercially-Available Bagged, Gunpowder, and Matcha Green Teas

**DOI:** 10.1007/s11130-023-01121-2

**Published:** 2023-11-03

**Authors:** Bailey R. Meyer, Haley M. White, Jared D. McCormack, Emily D. Niemeyer

**Affiliations:** Department of Chemistry and Biochemistry, Southwestern University, 1001 E. University Ave, Georgetown, TX 78626 USA

**Keywords:** *Camellia sinensis*, Green tea, Matcha, Antioxidant properties, Catechin content

## Abstract

The leaves of the *Camellia sinensis* plant are used to produce many different types of teas, and green tea is particularly noted for its health promoting properties which are attributed to high concentrations of phenolic compounds known as catechins. Green tea is available in forms such as bagged, gunpowder (rolled leaf), and matcha, a fine powder. This study evaluated the phenolic content, catechin composition, caffeine concentration, and antioxidant properties of fifteen commercially-available green teas with an emphasis on identifying differences between ceremonial and culinary matcha. Gunpowder and bagged green teas had higher total phenolic contents and greater CUPRAC (cupric ion reducing antioxidant capacity) and ORAC (oxygen radical absorbance capacity) antioxidant capacities than matcha teas. Among matcha samples, less expensive culinary teas had greater total phenolic contents and higher antioxidant capacities than ceremonial products. Hierarchical cluster analysis was used to determine similarities and differences in catechin composition and revealed that bagged and gunpowder green teas had the highest levels of the stereoisomers epi/catechin gallate, which correlated with their strong antioxidant properties. Bagged and gunpowder teas were the lowest cost products in this study, indicating that they are an economical choice for consumers who want a green tea beverage with high catechin content, although they generally had lower caffeine levels than matcha.

**Supplementary Information:**

The online version contains supplementary material available at 10.1007/s11130-023-01121-2.

## Introduction

Tea is produced from the leaves of the *Camellia sinensis* plant, and consumption of this well-known beverage is associated with the prevention of disease, particularly some forms of cancer [1]. The health benefits of tea are attributed to the presence of polyphenolic compounds [1], substances with strong antioxidant properties that can moderate the effects of oxidative damage within the body. The concentration and identity of bioactive phenolics in tea are greatly affected by the methods used to process the leaves [2]. For example, green tea is unfermented and deactivation of leaf enzymes during processing minimizes oxidation of the most prominent tea phenolics, flavonoids known as catechins. As a result, green tea is noted for its high catechin concentrations relative to other tea varieties such as white, oolong, and black [3].

Numerous types of green teas are available commercially, and they differ based on their country of origin as well as the methods used to cultivate the tea plant and process the leaves [2]. Most commonly, tea plants are grown in direct sunlight, the leaves are harvested and dried, then the tea is slightly ground and bagged or whole tea leaves can be rolled to produce gunpowder style tea. Matcha is a form of green tea, however, that is shade grown prior to harvest, and the dried tea leaves are ground into a fine powder [4]. Ceremonial grade is considered the highest quality matcha for use in the preparation of tea beverages, while culinary-grade matcha is more economical and may be used for drinks or as an ingredient in snacks or desserts.

Despite the increasing global availability and popularity of matcha, research regarding this form of green tea remains limited [4] and few studies have examined how the quality of matcha powder affects its phenolic composition. The aim of the current research was therefore to analyze the phenolic content, catechin composition, and antioxidant properties of commercially-available green tea varieties with an emphasis on evaluating differences between ceremonial and culinary matcha. Determining the levels of phenolic compounds in matcha is challenging because unlike other forms of green tea, it is traditionally consumed as a slurry rather than an extract. This issue has been previously addressed [5] by using a solvent that efficiently extracts phenolic compounds to determine the total amount of catechin that is available to consumers in matcha beverages. In this study, fifteen bagged, gunpowder, and matcha tea products (Table 1) were therefore extracted using acidified methanol since aqueous organic solvents optimize the recovery of phenolic compounds from tea [6]. This approach allows for a direct comparison of the concentrations of bioactive compounds found within these different forms of green tea as well as an evaluation of their antioxidant properties. Although catechins differ in their bioavailability and pharmacokinetic properties [7], research suggests that plasma antioxidant capacity increases linearly with ingestion of these green tea compounds [8]. Identifying commercial tea products with the highest catechin content may therefore allow consumers to prepare foods and beverages that most effectively protect against oxidative damage in the body and optimize potential health benefits.

**Table 1:** Green tea products in this study including manufacturer, origin, and cost (in USD per g of tea)

Product	Manufacturer	Origin	Cost (USD/g)
Green tea			
365 Everyday Value Green Tea (bagged)	Whole Foods Market	China	$0.03
Allegro Tea Himalayan Green (bagged)	Whole Foods Market	Southeast Asia	$0.08
Lipton Green Tea (bagged)	Unilever	Asia, India, Africa	$0.06
Pure Leaf Gunpowder Green Tea	Unilever	Indonesia	$0.04
Twinings of London Green Tea (bagged)	Twinings of London	Asia, India, Africa	$0.09
Culinary matcha			
Jade Leaf Matcha Green Tea Powder	Jade Leaf	Japan	$0.33
Kenkō Tea Matcha Green	Kenkō Tea	Japan	$0.37
Kiss Me Organics Matcha Green Tea Powder	Kiss Me Organics	Japan	$0.22
Matcha Wellness Green Tea Powder	Matcha Wellness	Japan	$0.33
Zen Spirit Matcha Tea	Zen Spirit	Japan	$0.30
Ceremonial Matcha			
Akira Matcha	Matcha Konomi	Japan	$0.83
Jade Leaf Matcha Green Tea Powder	Jade Leaf	Japan	$0.83
Kenkō Tea Matcha Green	Kenkō Tea	Japan	$0.93
Kiss Me Organics Ceremonial Matcha Green Tea Powder	Kiss Me Organics	Japan	$0.95
Matcha Organics Japanese Matcha	Matcha Organics	Japan	$0.87

## Materials and Methods

The Materials and Methods are included in the Supplementary Material for this article.

## Results and Discussion

### Analysis of Total Phenolic Content

**Fig. 1:**
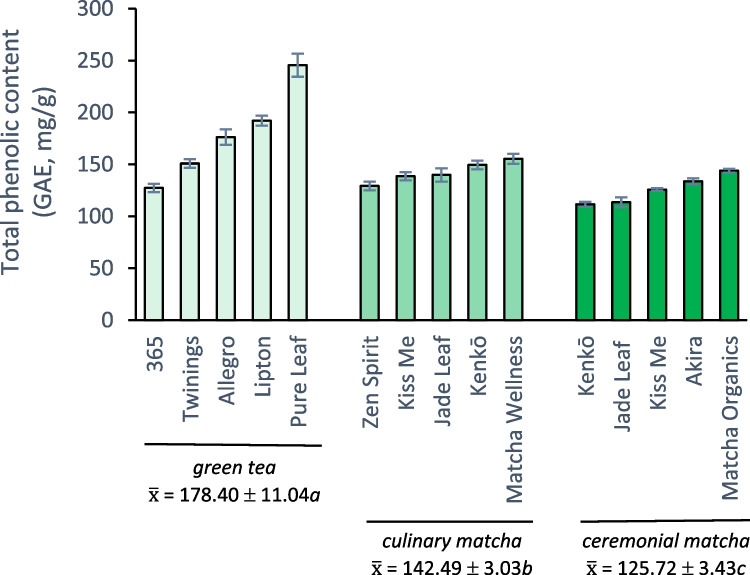
Average total phenolic contents (± standard error) reported in gallic acid equivalents (GAE, mg/g tea) for green tea and matcha products; average values ($\overline{\mathrm{x} })$ with the same letter are not statistically different

The total phenolic content (TPC) was measured for the green teas in this study (Fig. 1) using the Folin-Ciocalteu assay, which quantifies the total concentration of phenolic compounds while providing an estimate of the reducing capacity of each sample [9]. Total phenolic contents were statistically different among tea samples (*p* < 0.001) with Kenkō ceremonial matcha having the lowest TPC value (111.6 gallic acid equivalents, GAE, mg/g tea) while Pure Leaf gunpowder had the highest (245.5 GAE mg/g). Comparison of our results to other studies in the literature is complicated by the range of solvents and conditions used for the extraction of tea samples and the ways that TPC values are reported for tea (i.e., per volume of tea extract versus mass of dried leaf). However, Koláčková et al. [5] analyzed twelve matcha brands using an aqueous methanol extraction solvent (acidified aqueous methanol was used in the current study), and the authors reported a similar range of TPC values for their samples, 169 to 273 GAE mg/g tea.

Pure eaf gunpowder had significantly higher total phenolic content than all other tea samples except for the Lipton bagged (Supplementary Material, Fig. S1). The form of green tea had a significant effect on TPC values, with gunpowder and bagged teas having higher average TPC values (178.40 GAE mg/g) than both culinary (142.49 GAE mg/g) and ceremonial (125.7 GAE mg/g) matcha. Previous studies comparing the total phenolic content of bagged or leaf green tea with matcha have yielded conflicting results: matcha had a higher TPC than bagged Sencha green tea prior to a simulated digestion experiment [10] yet traditional green tea was also reported to have a higher TPC value than Tencha, the tea used to produce matcha [11]. These studies, however, compared only one sample of each tea type which may have obscured actual differences, since we observed significant variations in TPC values even among green tea and matcha of the same type (Supplementary Material, Fig. S1). For matcha samples of the same brand, culinary matcha consistently had a higher total phenolic content than its ceremonial counterpart. Kenkō culinary matcha, for example, had a significantly greater TPC value (149.5 GAE mg/g) than Kenkō ceremonial matcha (111.6 GAE mg/g), and a similar trend was observed for Kiss Me and Jade Leaf brands. Many phenolic compounds are associated with bitter and astringent flavors in tea, so it is not surprising that ceremonial-grade matcha, which is prized for its milder taste, generally exhibits a lower total phenolic content than culinary matcha.

### Measurement of Antioxidant Properties

**Fig. 2:**
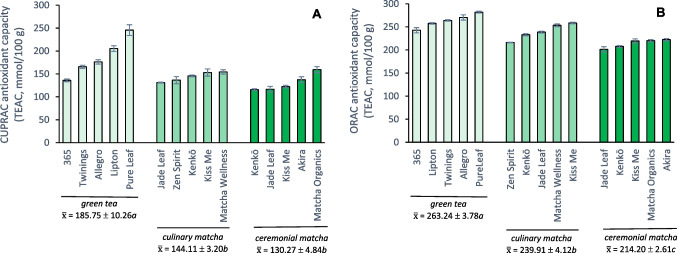
Average CUPRAC (**A**) and ORAC (**B**) antioxidant capacities (± standard error) reported as trolox equivalent antioxidant capacities (TEAC, mmol/100 g tea) for green tea and matcha products; average values ($\overline{\mathrm{x} })$ with the same letter on a given plot are not statistically different

Phenolic compounds within green tea are known to have strong antioxidant properties. The antioxidant capacities of the tea samples in this study were therefore measured using both the CUPRAC and ORAC methods and results are shown in Fig. 2. The CUPRAC assay utilizes an electron transfer (ET) mechanism [12] while the ORAC assay is based on hydrogen atom transfer (HAT) [9]. These assays measure complementary aspects of antioxidant activity since the ET-based CUPRAC assay estimates the reducing capacity of a sample while the HAT-based ORAC assay measures the ability of a sample to scavenge free radicals [9].

Pure Leaf gunpowder green tea had the highest CUPRAC antioxidant capacity (245.5 TEAC mmol/100 g), while Kenkō ceremonial matcha had the lowest (115.6 TEAC mmol/100 g). Few comparison values exist in the literature for CUPRAC antioxidant capacities of green tea, but an ethanolic extract of Twinings of London green tea was reported to have a CUPRAC value of ~ 125 mmol/100 g [13] which is slightly lower than the value we determined for Twinings bagged green tea, 164.6 mmol/100 g, but these differences are likely due to variations in extraction solvents and conditions. CUPRAC antioxidant capacities were statistically different among tea products (*p* < 0.001) with Pure Leaf gunpowder having significantly greater CUPRAC values than all other products except Lipton green tea (Supplementary Material, Fig. S2). Average CUPRAC antioxidant capacities for bagged/gunpowder green teas (185.75 ± 10.26 TEAC mmol/100 g) were significantly higher than the values for culinary (*p* < 0.001) and ceremonial (*p* < 0.001) matcha.

ORAC values ranged from 201.2 to 281.8 TEAC mmol/100 g with Jade Leaf ceremonial matcha having the lowest ORAC antioxidant capacity while Pure Leaf gunpowder had the highest. Although ORAC values in the current study were higher than some reports in the literature, Sharpe et al. [14] found a large range of ORAC antioxidant capacities (from ~ 100 to 350 TEAC mmol/100 g) for 24 green tea products subjected to six successive hot water extractions and, interestingly, they also noted that a loose-leaf gunpowder product had one of the highest ORAC values among their samples. In the current work, the ORAC antioxidant capacity of Pure Leaf gunpowder was significantly greater than all other green tea products except Allegro bagged tea (270.3 TEAC mmol/100 g; Supplementary Material, Fig. S3). The average ORAC antioxidant capacities of gunpowder/bagged green teas (263.24 ± 3.78 TEAC mmol/100 g) were statistically higher than the values for culinary matcha (239.91 ± 4.12 TEAC mmol/100 g; *p* < 0.001) and ceremonial matcha (214.20 ± 2.61 TEAC mmol/100 g; *p* < 0.001).

Comparing the two assays, ORAC had higher antioxidant capacity values than CUPRAC for every tea sample. Although the magnitude of the measured antioxidant capacities differed, ORAC and CUPRAC values had a significant (*p* < 0.001) strong (*R* = 0.763) correlation, with tea products having the highest antioxidant capacities in one assay generally exhibiting high values for the other. For example, four teas – Pure Leaf, Allegro, Twinings, and Lipton – were among the samples with the five highest antioxidant capacities for both assays. Similarly, products with weaker antioxidant properties, such as the ceremonial matchas made by Jade Leaf, Kenkō, and Kiss Me, were among the five lowest antioxidant capacities for both assays.

When comparing teas of the same brand, Kiss Me, Jade Leaf, and Kenkō culinary matchas had higher ORAC and CUPRAC antioxidant capacities than their ceremonial counterparts. This result is likely due to the fact that culinary-grade matcha generally has a higher total phenolic content than ceremonial matcha tea. In fact, CUPRAC antioxidant capacities had a very strong (*R* = 0.958) significant (*p* < 0.001) correlation with total phenolic content values. Because the Folin-Ciocalteu and CUPRAC assays both rely on redox-based ET mechanisms, it is expected that they would have a high degree of correlation [9]. The correlation between ORAC antioxidant capacities and TPC values was also significant (*p* < 0.001) but the correlation was weaker (*R* = 0.764).

### Determination of Catechin and Caffeine Content

We investigated the concentrations of the most prevalent catechins within our green teas and results are presented in Table 2 (Supplementary Material, Figs. S4-S8 summarize significant differences in catechin contents among samples). Epigallocatechin gallate (EGCG) and epigallocatechin (EGC) were the predominant catechins, with EGCG present at highest concentration within all tea samples in the study. EGCG is recognized for its health-promoting properties [15], and sample concentrations ranged from 23.29 mg/g for 365 bagged green tea to 70.22 mg/g for Pure Leaf gunpowder. EGCG concentrations were statistically different among tea samples (*p* < 0.001) with 365 having significantly lower EGCG values than all other teas while Pure Leaf gunpowder had higher EGCG concentrations than all samples except Kenkō culinary matcha, Akira ceremonial matcha, and Matcha Organics ceremonial matcha (Supplementary Material, Fig. S4). Average EGCG concentrations for bagged and gunpowder green tea (46.14 ± 4.24 mg/g) were similar to values previously reported for boiling water extracts of nine Chinese green teas, 34 to 58 mg/g [3]. Culinary matcha (50.53 ± 2.41 mg/g) and ceremonial matcha (56.57 ± 2.19 mg/g) had average EGCG concentrations that were similar in magnitude but slightly lower overall than Horie et al. [16] reported for acidified ethanolic extracts of culinary and ceremonial matcha, 53 to 93 mg/g. Although we found that bagged and gunpowder green teas had the lowest EGCG concentrations and ceremonial matcha teas had the highest, differences across tea types were not statistically significant.

**Table 2:** Average catechin and caffeine concentrations (± standard error; all reported in mg/g tea) for bagged and gunpowder green tea, culinary matcha, and ceremonial matcha samples

Brand	Epicatechin	EGC	EGCG	GCG	E-CG	Caffeine
Green tea						
365	6.00 ± 0.18	13.86 ± 0.25	23.29 ± 1.15	2.06 ± 0.11	9.54 ± 0.53	17.11 ± 0.46
Allegro	6.50 ± 0.51	19.47 ± 1.20	46.24 ± 3.41	2.69 ± 0.31	16.06 ± 0.99	19.96 ± 1.96
Lipton	8.17 ± 0.22	24.38 ± 1.20	52.96 ± 1.08	2.36 ± 0.15	17.37 ± 0.71	23.52 ± 0.57
PureLeaf	7.26 ± 0.57	23.83 ± 1.04	70.22 ± 2.85	1.10 ± 0.15	28.02 ± 0.98	32.38 ± 3.37
Twinings	7.21 ± 0.37	19.57 ± 0.27	38.01 ± 0.38	3.16 ± 0.49	12.92 ± 1.57	22.45 ± 1.28
Culinary matcha						
Jade Leaf	9.36 ± 0.66	38.04 ± 1.97	47.95 ± 3.05	2.52 ± 0.34	8.80 ± 1.07	17.74 ± 2.12
Kenkō	5.56 ± 0.31	30.74 ± 0.88	62.15 ± 1.63	1.26 ± 0.20	10.77 ± 0.51	32.23 ± 0.78
Kiss Me	11.21 ± 0.75	29.18 ± 1.79	36.70 ± 2.43	2.84 ± 0.32	9.31 ± 0.41	18.48 ± 1.11
Matcha Wellness	4.34 ± 0.34	19.75 ± 0.63	51.63 ± 1.02	ND^a^	11.89 ± 0.03	33.45 ± 1.09
Zen Spirit	4.76 ± 0.33	25.20 ± 1.11	54.21 ± 3.25	ND^a^	7.14 ± 0.40	29.57 ± 2.85
Ceremonial matcha						
Akira	5.04 ± 0.44	26.21 ± 0.86	60.40 ± 1.89	0.73 ± 0.37	7.66 ± 0.50	34.89 ± 2.44
Jade Leaf	4.22 ± 0.33	21.34 ± 1.30	49.03 ± 2.95	1.43 ± 0.15	6.08 ± 0.65	24.95 ± 2.52
Kenkō	3.75 ± 0.79	17.91 ± 0.54	49.70 ± 1.59	1.47 ± 0.24	6.09 ± 0.21	31.55 ± 1.62
Kiss Me	4.17 ± 0.52	22.71 ± 0.16	53.98 ± 1.60	1.31 ± 0.07	5.12 ± 0.12	30.87 ± 0.56
Matcha Organics	5.25 ± 0.20	24.47 ± 0.11	69.73 ± 1.34	1.96 ± 0.25	9.21 ± 0.46	35.01 ± 2.46

*EGC* epigallocatechin; *EGCG* epigallocatechin gallate; *GCG* gallocatechin gallate and *E-CG* epi/catechin gallate

^a^ *ND* not detected

Epigallocatechin concentrations ranged from 13.86 mg/g for 365 bagged tea to 38.04 mg/g for Jade Leaf culinary matcha. EGC values were statistically different among tea samples (*p* < 0.001) with Jade Leaf culinary matcha having a significantly higher concentration than all other samples, and 365 tea having significantly lower concentrations than all teas except Kenkō ceremonial matcha (Supplementary Material, Fig. S5). EGC concentrations for teas in this study were in good agreement with those reported by Horie et al. [16] for matcha products sold in the United States, 10 to 46 mg/g. The form of green tea significantly affected EGC concentrations (*p* < 0.001), with culinary matcha having statistically higher EGC values (28.58 ± 1.70 mg/g) than both bagged/gunpowder green tea (20.22 ± 1.07 mg/g) and ceremonial matcha (22.52 ± 0.81 mg/g). This is a particularly interesting result since it differs from previous research. Green tea was reported to have higher EGC content than Tencha leaves (used to produce matcha) grown in high-shade conditions [11] while ceremonial matcha has been noted to have higher EGC values than culinary matcha [16].

Caffeine concentrations were also quantified within the fifteen tea products in this study (Table 2). Caffeine is a bioactive methylxanthine with stimulant properties and is produced by the tea plant throughout its growth. Although statistically significant differences in caffeine content were not found among tea products, concentrations varied from 17.11 mg/g for 365 bagged green tea to 35.01 mg/g for Matcha Organics ceremonial matcha. Caffeine concentrations for tea products in this study are similar to those in the literature: Astill et al. [17] reported that an aqueous green tea extract had a caffeine concentration of 26.9 mg/g, and Koláčková et al. [5] found that methanolic extracts of twelve matcha products had caffeine contents ranging from 14.4 to 34.1 mg/g. Notably, ceremonial matcha products had greater average caffeine content (31.45 ± 1.08 mg/g) than culinary matcha (26.29 ± 1.86 mg/g) and significantly higher caffeine concentrations than bagged/gunpowder green tea (23.08 ± 1.43 mg/g; *p* = 0.002). Previous research on tea clones suggests that caffeine concentrations in tea leaves decrease with age [18], and our results show that ceremonial matcha, the highest-quality and most expensive product which is most likely to be produced from the youngest tea leaves, had the highest caffeine content among teas in this study.

### Catechin Profiles of Green Tea Products

**Fig. 3:**
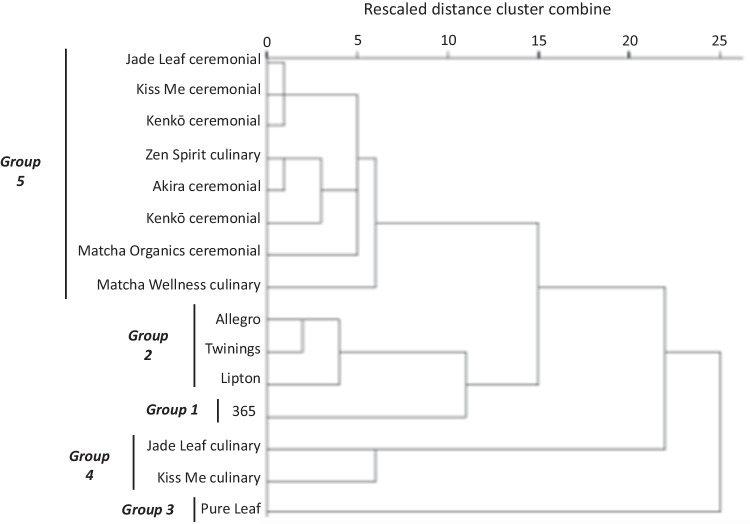
Dendrogram plot displaying clustering of the green tea and matcha products in this study based on catechin concentrations

Hierarchical cluster analysis was used to group tea samples based on similarities in their catechin profiles (Fig. 3) and resulted in a five-cluster solution. The first cluster, Group 1, contained a single sample, 365 bagged green tea, that was characterized by low concentrations of all catechins compared to other groups. The next cluster, Group 2, contained all of the other bagged green teas in the study (Allegro, Twinings, and Lipton) and had relatively low concentrations of epicatechin, epigallocatechin (EGC), and EGCG compared to other clusters, but had the highest concentration of gallocatechin gallate (GCG) and high concentrations of the stereoisomers epi/catechin gallate (E-CG). Group 3 contained one sample, Pure Leaf gunpowder tea, and was characterized by the highest EGCG and E-CG concentrations among clusters. The next cluster, Group 4, contained two culinary matchas, Jade Leaf and Kiss Me, and had the highest epicatechin and EGC concentrations among clusters. The last cluster, Group 5, was the largest and contained eight samples, all five ceremonial matchas in the study and two culinary matchas. Group 5 had the lowest epicatechin, GCG, and E-CG concentrations of all clusters but EGC and EGCG concentrations were similar to the other groups.

Pure Leaf gunpowder was in a cluster characterized by the highest EGCG (70.22 mg/g) and E-CG (28.02 mg/g) concentrations among tea products, and notably Pure Leaf also had the highest CUPRAC and ORAC antioxidant capacities. Group 2, which contained Allegro, Twining, and Lipton bagged green teas, had moderate EGCG concentrations (average = 45.74 mg/g) but relatively high E-CG levels (average = 15.45 mg/g), and these products had among the five highest CUPRAC and ORAC antioxidant capacities. Group 5, which contained all of the ceremonial matchas and three culinary matchas, had higher EGCG levels than Group 2 (average = 56.35 mg/g), contained the lowest E-CG content (8.00 mg/g) among all clusters, and had the lowest ORAC and CUPRAC values in this study.

Although epi/catechin gallate was found within green tea samples at lower concentrations overall than both EGCG and epigallocatechin, E-CG concentrations had strong significant correlations with both CUPRAC (*R* = 0.942; *p* < 0.001) and ORAC (*R* = 0.800; *p* < 0.001) antioxidant capacities. In contrast, EGCG concentrations had a much lower correlation with CUPRAC values (*p* = 0.014; *R* = 0.365) and exhibited no correlation with ORAC antioxidant capacities. Among green tea catechins, EGCG is known to be one of the strongest free radical scavengers due to its chemical structure [15] yet the reported ORAC antioxidant capacity for EGCG, 8.2 mmol TEAC/mmol catechin, is lower than that of epicatechin gallate, 10.4 mmol TEAC/mmol catechin [19]. Together, these results suggest that the greater antioxidant properties that we observe for green teas in Groups 2 and 3 relate to the amount of the epi/catechin gallate stereoisomers within these products, even though other catechins such as EGCG and EGC may be present at much higher concentrations.

A variety of factors are known to affect the phenolic composition of tea [17]. The *Camellia sinensis* plants used to produce the green and matcha tea products in this study were grown in different geographical regions (Table 1) and were therefore subject to varying agronomic conditions during their development. We found that Pure Leaf gunpowder, the only tea product grown in Indonesia, had the highest total phenolic content as well as the highest EGCG and E-CG levels. Additionally, Pure Leaf gunpowder was the only loose-leaf tea within this study, and our results confirmed other research suggesting loose-leaf green teas contain higher phenolic content than bagged varieties [20]. All of the matcha products originated from the same country, Japan, and hierarchical cluster analysis revealed most of these teas had a similar catechin profile regardless of their rated quality as culinary or ceremonial. The exception was two culinary matchas, Jade Leaf and Kiss Me, that had higher epicatechin and EGC concentrations along with slightly lower EGCG concentrations than all other matcha products.

The green teas in Groups 2 and 3 were also among the lowest cost products within this study (Table 1). Pure Leaf gunpowder had the highest total phenolic content, ORAC and CUPRAC antioxidant capacities, EGCG levels, and E-CG concentrations, yet was one of the lowest cost tea products, $0.04 USD/g. Similarly, Group 2 contained all of the bagged green tea products except 365 Everyday Value and had relatively high catechin levels (particularly epi/catechin gallate) as well as high ORAC and CUPRAC values, but maintained a relatively low average cost of $0.08 USD/g. In contrast, the five ceremonial and three culinary matchas contained in Group 5 had a much higher average cost of $0.68 USD/g, but contained relatively low levels of most catechins except EGCG, and were characterized by low total phenolic content and ORAC and CUPRAC antioxidant capacities.

## Conclusions

Gunpowder and bagged green teas generally exhibited higher total phenolic contents and greater antioxidant properties than culinary and ceremonial matchas. EGCG and epigallocatechin were the predominant catechins found in all tea samples, and most green tea products (except 365 bagged tea) contained comparable levels of EGCG. Hierarchical cluster analysis revealed that some of the lowest cost green teas – such as Pure Leaf gunpowder and Allegro, Twinings, and Lipton bagged teas – had particularly high levels of the stereoisomers epi/catechin gallate which correlated with high measured ORAC and CUPRAC antioxidant capacities. Results also demonstrated that less expensive culinary-grade matcha green teas had higher total phenolic contents and antioxidant capacities when compared to higher cost ceremonial-grade matcha.

A variety of factors ultimately affect the levels of phenolic compounds, catechins, and antioxidants that a consumer receives from a green tea beverage or snack. The nutritional value of green tea may vary due to agronomic practices, manufacturing techniques, methods the consumer uses to prepare their food or drink, as well as differences in the absorption and metabolism of green tea phenolics which can occur across individuals [7]. We examined methanolic extracts of commercially-available green teas to determine the total amounts of phenolic compounds and catechins found within these products. Results showed that most of the bagged and gunpowder green teas in this study contained high levels of catechins such as the epi/catechin gallate isomers, which correlated with these products having greater antioxidant properties. With the lower overall cost of bagged and gunpowder teas, these products are an economical choice for individuals who are interested in purchasing green tea with a high catechin content, although their caffeine levels were slightly lower than matcha. Identifying green teas with high catechin concentrations provides consumers with important knowledge that they may use to select products which are rich in antioxidants. Regular dietary consumption of phenolic antioxidants is associated with a variety of health benefits including the prevention of diseases associated with oxidative stress.

### Supplementary Information

Below is the link to the electronic supplementary material.

Supplementary file1 (DOCX 45.4 KB)

## Data Availability

Datasets from the current study are available from the corresponding author upon request.
