# Supplementary material for: Catechin Composition, Phenolic Content, and Antioxidant Properties of Commercially-Available Bagged, Gunpowder, and Matcha Green Teas
Source: Plant Foods Hum Nutr. 2023 Nov 3;78(4):662–9. doi: 10.1007/s11130-023-01121-2 (PMC10665233; doi:10.1007/s11130-023-01121-2)
Supplement: Supplementary file 1 — Supplementary file1 (DOCX 45.4 KB) [file 11130_2023_1121_MOESM1_ESM.docx]

Supplementary Material

**Catechin composition, phenolic content, and antioxidant properties of**

**commercially-available bagged, gunpowder, and matcha green teas**

Bailey R. Meyer, Haley M. White, Jared D. McCormack, and Emily D. Niemeyer

**Materials and Methods**

*Chemicals and Reagents*

Analytical standards such as gallic acid, caffeine, and trolox (6-hydroxy-2,5,7,8-tetramethylchroman-2-carboxylic acid) were of the highest available purity and were purchased from MilliporeSigma (St. Louis, MO, USA). Green tea catechin analytical standards (epicatechin, catechin gallate, epicatechin gallate, epigallocatechin, gallocatechin gallate, and epigallocatechin gallate) were purchased from INDOFINE Chemical Co. (Somerville, NJ, USA). Folin­Ciocalteu phenolic reagent was obtained from VWR (Radnor, PA, USA). Reagents such as neocuproine, copper (II) chloride, ammonium acetate, sodium carbonate, 2,2’-azobis(2-amidinopropane) dihydrochloride (AAPH), sodium fluorescein, sodium hydrogen phosphate, and hydrochloric acid were purchased from MilliporeSigma. Formic acid (puriss ACS reagent grade) and acetonitrile (HPLC, high-performance liquid chromatography, grade) were also obtained from MilliporeSigma. HPLC-grade methanol and ethanol were purchased from Pharmco-AAPER (Brookfield, CT, USA).

*Sample Preparation*

Fifteen green tea products sold by ten different companies were purchased from an online retailer (Amazon.com, Seattle, WA). Samples consisted of four bagged teas, one gunpowder rolled whole leaf tea, five culinary-grade matcha teas, and five ceremonial-grade matcha teas. Tea samples were frozen immediately upon receipt and kept in dark storage in their original packaging prior to extraction. Three replicates were extracted for each of the 15 tea products (*n* = 45 total samples) using acidified methanol as the solvent and a method adapted from Zuo et al. [1]. Briefly, 0.10-0.15 g of each tea sample was weighed and the mass recorded. Samples were each sonicated in 0.75 mL of acidified methanol (0.15% hydrochloric acid in 80% aqueous methanol solution) for 45 min, centrifuged at 13,200 RPM for 15 min, and the supernatant was removed. The extraction procedure was repeated on the remaining pellet, and the combined supernatants were saved as the final sample extracts and kept in dark storage at -80°C prior to analysis.

*Total Phenolic Content Determination*

The total phenolic contents of all tea samples were measured using a Folin-Ciocalteu assay [2] that was modified for a microplate reader. Tea sample or gallic acid standard (100 µL) was combined with deionized water (150 µL), Folin-Ciocalteu reagent (125 µL), and 20% aqueous sodium carbonate (625 µL) in the order stated. The solution was incubated at room temperature for 20 min, centrifuged at 13,200 RPM for 5 min, then the supernatant was pipetted onto a 96-well plate. The absorbance of all sample and standard mixtures was measured at 735 nm using a BioRad Benchmark Plus microplate spectrophotometer (Hercules, CA, USA) against a blank containing water (150 µL), acidified methanol (100 µL), and 20% aqueous sodium carbonate (625 µL). A gallic acid standard curve ranging in concentration from 12.5 to 800.0 mg/L was used to quantify the sample absorbance values, and final total phenolic contents for all tea samples were reported as gallic acid equivalents (GAE) in mg/g tea.

*Analysis of Tea Catechins and Caffeine*

High-performance liquid chromatography (HPLC) was used to identify and quantify individual catechin and caffeine levels within all tea samples. The method utilized a tri-pump Shimadzu HPLC system (Columbia, MD, USA) with an SIL-20AHT autosampler, diode array detector, and Agilent (Santa Clara, CA, USA) Zorbax SB-C_18_ column (1.8 µm, 4.6 $\times$ 50 mm). Separation conditions were based on a previously described method [3] and consisted of a gradient elution with 0.2% aqueous formic acid (eluent A) and 0.1% formic acid in acetonitrile (eluent B) at a flow rate of 1 mL/min. The sample injection volume was 10 µL and the following gradient was used: 0-8 min, 0-5% B; 8-10 min, 5-25% B; 10-22 min, 25-100% B; 22-25 min, 100-5% B; 25-25.1 min, 5-0% B; 25.1-30 min, 0% B. Detection occurred by collecting absorbance spectra from 200 to 300 nm.

Individual catechins and caffeine were monitored at 230 nm and identified in tea samples based on characteristic retention times (epigallocatechin = 12.928 ± 0.017 min; caffeine = 13.271 ± 0.018 min; epicatechin = 13.505 ± 0.008 min; epigallocatechin gallate = 13.573 ± 0.008 min; gallocatechin gallate = 13.683 ± 0.006 min; epi/catechin gallate = 14.120 ± 0.006 min), associated absorbance spectra, and comparison to analytical standards. Catechins and caffeine were quantified in tea samples by comparing the integrated peak areas for individual compounds to calibration curves prepared over a wide linear (*R^2^* ≥ 0.99) concentration range (1.17 – 75.00 mg/L), with final analyte concentrations reported in mg/g tea.

*Measurement of Antioxidant Capacities*

Antioxidant capacities were determined using both the cupric reducing antioxidant capacity (CUPRAC) and the oxygen radical absorbance capacity (ORAC) assays. For the CUPRAC assay, the method of Apak et al. [4] was adapted for a microplate reader. Solutions of 1.0 x 10^-2^ M copper(II) chloride (300 µL), 1.0 M ammonium acetate buffer at pH 7.0 (300 µL), and 7.5 mM neocuproine in 96% aqueous ethanol (300 µL) were mixed in a black microfuge tube then 150 µL of tea sample, trolox standard, or deionized water (control) was added. Solutions were incubated at room temperature for 30 min, pipetted onto a 96-well microplate, and the sample and standard absorbance values were measured at 450 nm against the control using a BioRad Benchmark Plus microplate spectrophotometer. CUPRAC values were determined by comparing sample absorbances to a trolox calibration curve (20.0 to 800.0 mg/L), and final concentrations were calculated as trolox equivalent antioxidant capacities (TEAC) in mmol/100 g tea.

For the ORAC assay, a high-throughput method developed by Huang et al. [5] was used with modifications. A sodium fluorescein solution (150 µL; 8 nM in 75 mM phosphate buffer, pH = 7.4) was pipetted onto a black 96-well microplate. A 25 µL aliquot of tea sample extract, trolox standard, or phosphate buffer (control) was added to each well, the plate was covered, and the mixture was incubated for 30 min at 37 °C. A 150 mM solution of AAPH in phosphate buffer (25 µL, prepared fresh daily) was then added to each well followed by shaking for 10 sec. A BioTek Synergy H1 microplate reader (Winooski, VT, USA) was used to kinetically monitor the sample and standard fluorescence (excitation = 485 nm; emission = 528 nm; 20 nm bandpass) versus the control with all samples, standards, and the control analyzed in triplicate. BioTek’s Gen5 data analysis software was used to calculate the fluorescence area under the curve (AUC) for collected kinetic data [6], and net AUC values were determined according to the following equation:

net AUC = AUC_sample_ – AUC_control_

where AUC_sample_ = sample (or trolox standard) area under the curve and AUC_control_ = control area under the curve. Tea sample net AUC values were compared to a trolox calibration curve ranging in concentration from 6.25 to 100.00 µM and final ORAC values were reported as TEAC in mmol/100 g tea.

*Statistical Analyses*

All values are reported as the mean ± standard error, calculated from replicate samples (*n* = 3 for each tea product). Statistical analyses of data sets were completed using IBM SPSS Statistics (SPSS Inc., Chicago, IL, USA). One-way analysis of variance (ANOVA) with the Tukey post-hoc test was used to determine if statistical differences existed among total phenolic contents, CUPRAC and ORAC antioxidant capacities, and individual catechin and caffeine concentrations for different tea products as well as tea types (e.g., gunpowder/bagged green tea, culinary-grade matcha, or ceremonial-grade matcha). Data sets were tested for normality using the Shapiro-Wilk test and homogeneity of variance using Levene’s test. Several data sets required a transformation (inverse, square root, or log) to achieve normality prior to ANOVA analysis. Normality could not be achieved for the caffeine concentration data set so a nonparametric Kruskall-Wallis test was used with pairwise comparisons and the Bonferroni correction applied. For data sets in which homogeneity of variance could not be achieved, the Brown-Forsythe test was used with the Dunnett T3 post-hoc test. For all statistical analyses, significance was determined the *p* < 0.05 level.

Tea samples were grouped using hierarchical cluster analysis (HCA) to identify similarities in the profiles of their individual catechin concentrations. The cluster analysis used between-groups linkage, Euclidian distance measurement intervals, and z-scores to standardize variables with final results visualized using a dendrogram plot.

Bivariate Pearson correlation analysis was used to measure whether a linear relationship existed between ORAC and CUPRAC antioxidant capacities; total phenolic contents and both ORAC and CUPRAC antioxidant capacities; epigallocatechin gallate concentrations and both ORAC and CUPRAC antioxidant capacities; and epi/catechin gallate concentrations and both ORAC and CUPRAC antioxidant capacities. A two-tailed test of statistical significance was used at the *p* < 0.05 level. The magnitude of the calculated *r* value was used to determine the strength of the correlation with 0.3–0.5 considered a moderate correlation while > 0.5 indicated a strong correlation between variables.

**References**

1. Zuo Y, Chen H, Deng Y (2002) Simultaneous determination of catechins, caffeine and gallic acids in green, oolong, black and pu-erh teas using HPLC with a photodiode array detector. Talanta 57:307–316. <https://doi.org/10.1016/S0039-9140(02)00030-9>
2. Singleton VL, Rossi Jr JA (1965) Colorimetry of total phenolics with phosphomolybdic–phosphotungstic acid reagents. Am J Enol Vitic 16:144–158. [https://doi.org/10.5344/ajev.1965.16.3.144](file:///Users/niemeyee/Documents/matcha%20tea%20-%20Haley,%20Bailey,%20and%20Jared/10.5344/ajev.1965.16.3.144)
3. Araya-Farias M, Alain G, Elodie R, Bazinet L (2014) Rapid HPLC-MS method for the simultaneous determination of tea catechins and folates. J Agric Food Chem 62:4241–4250. <https://doi.org/10.1021/jf4053258>
4. Apak R, Güçlü K, Özyürek M, Karademir SE (2004) Novel total antioxidant capacity index for dietary polyphenols and vitamins C and E, using their cupric ion reducing capability in the presence of neocuproine: CUPRAC method. J Agric Food Chem 52: 7970–7981. <https://doi.org/10.1021/jf048741x>
5. Huang D, Ou B, Hampsch-Woodill M, Flanagan JA, Prior RL (2002) High-throughput assay of oxygen radical absorbance capacity (ORAC) using a multichannel liquid handling system coupled with a microplate fluorescence reader in 96-well format. J Agric Food Chem 50:4437−4444. <https://doi.org/10.1021/jf0201529>
6. Brescia PJ (2012) Determination of antioxidant potential using an oxygen radical absorbance capacity (ORAC) assay with Synergy^TM^ H4 (Application Note). <https://www.agilent.com/cs/library/applications/determination-of-antioxidant-potential-5994-3310EN-agilent.pdf> Accessed 17 August 2023

|  |  | Green tea | | | | | Culinary | | | | | Ceremonial | | | | |
| --- | --- | --- | --- | --- | --- | --- | --- | --- | --- | --- | --- | --- | --- | --- | --- | --- |
|  |  | Allegro | 365 | Twinings | Pure Leaf | Lipton | Zen Spirit | Kenkō | Matcha Wellness | Jade Leaf | Kiss Me | Kiss Me | Kenkō | Akira | Jade Leaf | Matcha Organics |
| Green tea | Allegro |  |  |  |  |  |  |  |  |  |  |  |  |  |  |  |
|  | 365 |  |  |  |  |  |  |  |  |  |  |  |  |  |  |  |
|  | Twinings |  |  |  |  |  |  |  |  |  |  |  |  |  |  |  |
|  | Pure Leaf |  |  |  |  |  |  |  |  |  |  |  |  |  |  |  |
|  | Lipton |  |  |  |  |  |  |  |  |  |  |  |  |  |  |  |
| Culinary | Zen Spirit |  |  |  |  |  |  |  |  |  |  |  |  |  |  |  |
|  | Kenkō |  |  |  |  |  |  |  |  |  |  |  |  |  |  |  |
|  | Matcha Wellness |  |  |  |  |  |  |  |  |  |  |  |  |  |  |  |
|  | Jade Leaf |  |  |  |  |  |  |  |  |  |  |  |  |  |  |  |
|  | Kiss Me |  |  |  |  |  |  |  |  |  |  |  |  |  |  |  |
| Ceremonial | Kiss Me |  |  |  |  |  |  |  |  |  |  |  |  |  |  |  |
|  | Kenkō |  |  |  |  |  |  |  |  |  |  |  |  |  |  |  |
|  | Akira |  |  |  |  |  |  |  |  |  |  |  |  |  |  |  |
|  | Jade Leaf |  |  |  |  |  |  |  |  |  |  |  |  |  |  |  |
|  | Matcha Organics |  |  |  |  |  |  |  |  |  |  |  |  |  |  |  |

.

**Figure S1.** Statistical differences in average total phenolic content among green tea extracts. White denotes no statistical differences between samples while blue (0.05 > *p* < 0.01), green (0.01 > *p* <0.001), and orange (*p* < 0.001) indicate different levels of statistical significance.

|  |  | Green tea | | | | | Culinary | | | | | Ceremonial | | | | |
| --- | --- | --- | --- | --- | --- | --- | --- | --- | --- | --- | --- | --- | --- | --- | --- | --- |
|  |  | Allegro | 365 | Twinings | Pure Leaf | Lipton | Zen Spirit | Kenkō | Matcha Wellness | Jade Leaf | Kiss Me | Kiss Me | Kenkō | Akira | Jade Leaf | Matcha Organics |
| Green tea | Allegro |  |  |  |  |  |  |  |  |  |  |  |  |  |  |  |
|  | 365 |  |  |  |  |  |  |  |  |  |  |  |  |  |  |  |
|  | Twinings |  |  |  |  |  |  |  |  |  |  |  |  |  |  |  |
|  | Pure Leaf |  |  |  |  |  |  |  |  |  |  |  |  |  |  |  |
|  | Lipton |  |  |  |  |  |  |  |  |  |  |  |  |  |  |  |
| Culinary | Zen Spirit |  |  |  |  |  |  |  |  |  |  |  |  |  |  |  |
|  | Kenkō |  |  |  |  |  |  |  |  |  |  |  |  |  |  |  |
|  | Matcha Wellness |  |  |  |  |  |  |  |  |  |  |  |  |  |  |  |
|  | Jade Leaf |  |  |  |  |  |  |  |  |  |  |  |  |  |  |  |
|  | Kiss Me |  |  |  |  |  |  |  |  |  |  |  |  |  |  |  |
| Ceremonial | Kiss Me |  |  |  |  |  |  |  |  |  |  |  |  |  |  |  |
|  | Kenkō |  |  |  |  |  |  |  |  |  |  |  |  |  |  |  |
|  | Akira |  |  |  |  |  |  |  |  |  |  |  |  |  |  |  |
|  | Jade Leaf |  |  |  |  |  |  |  |  |  |  |  |  |  |  |  |
|  | Matcha Organics |  |  |  |  |  |  |  |  |  |  |  |  |  |  |  |

**Figure S2.** Statistical differences in average CUPRAC (cupric reducing antioxidant capacity) values among green tea extracts. White denotes no statistical differences between samples while blue (0.05 > *p* < 0.01), green (0.01 > *p* <0.001), and orange (*p* < 0.001) indicate different levels of statistical significance.

|  |  | Green tea | | | | | Culinary | | | | | Ceremonial | | | | |
| --- | --- | --- | --- | --- | --- | --- | --- | --- | --- | --- | --- | --- | --- | --- | --- | --- |
|  |  | Allegro | 365 | Twinings | Pure Leaf | Lipton | Zen Spirit | Kenkō | Matcha Wellness | Jade Leaf | Kiss Me | Kiss Me | Kenkō | Akira | Jade Leaf | Matcha Organics |
| Green tea | Allegro |  |  |  |  |  |  |  |  |  |  |  |  |  |  |  |
|  | 365 |  |  |  |  |  |  |  |  |  |  |  |  |  |  |  |
|  | Twinings |  |  |  |  |  |  |  |  |  |  |  |  |  |  |  |
|  | Pure Leaf |  |  |  |  |  |  |  |  |  |  |  |  |  |  |  |
|  | Lipton |  |  |  |  |  |  |  |  |  |  |  |  |  |  |  |
| Culinary | Zen Spirit |  |  |  |  |  |  |  |  |  |  |  |  |  |  |  |
|  | Kenkō |  |  |  |  |  |  |  |  |  |  |  |  |  |  |  |
|  | Matcha Wellness |  |  |  |  |  |  |  |  |  |  |  |  |  |  |  |
|  | Jade Leaf |  |  |  |  |  |  |  |  |  |  |  |  |  |  |  |
|  | Kiss Me |  |  |  |  |  |  |  |  |  |  |  |  |  |  |  |
| Ceremonial | Kiss Me |  |  |  |  |  |  |  |  |  |  |  |  |  |  |  |
|  | Kenkō |  |  |  |  |  |  |  |  |  |  |  |  |  |  |  |
|  | Akira |  |  |  |  |  |  |  |  |  |  |  |  |  |  |  |
|  | Jade Leaf |  |  |  |  |  |  |  |  |  |  |  |  |  |  |  |
|  | Matcha Organics |  |  |  |  |  |  |  |  |  |  |  |  |  |  |  |

**Figure S3.** Statistical differences in average ORAC (oxygen reducing antioxidant capacity) values among green tea extracts. White denotes no statistical differences between samples while blue (0.05 > *p* < 0.01), green (0.01 > *p* <0.001), and orange (*p* < 0.001) indicate different levels of statistical significance.

|  |  | Green tea | | | | | Culinary | | | | | Ceremonial | | | | |
| --- | --- | --- | --- | --- | --- | --- | --- | --- | --- | --- | --- | --- | --- | --- | --- | --- |
|  |  | Allegro | 365 | Twinings | Pure Leaf | Lipton | Zen Spirit | Kenkō | Matcha Wellness | Jade Leaf | Kiss Me | Kiss Me | Kenkō | Akira | Jade Leaf | Matcha Organics |
| Green tea | Allegro |  |  |  |  |  |  |  |  |  |  |  |  |  |  |  |
|  | 365 |  |  |  |  |  |  |  |  |  |  |  |  |  |  |  |
|  | Twinings |  |  |  |  |  |  |  |  |  |  |  |  |  |  |  |
|  | Pure Leaf |  |  |  |  |  |  |  |  |  |  |  |  |  |  |  |
|  | Lipton |  |  |  |  |  |  |  |  |  |  |  |  |  |  |  |
| Culinary | Zen Spirit |  |  |  |  |  |  |  |  |  |  |  |  |  |  |  |
|  | Kenkō |  |  |  |  |  |  |  |  |  |  |  |  |  |  |  |
|  | Matcha Wellness |  |  |  |  |  |  |  |  |  |  |  |  |  |  |  |
|  | Jade Leaf |  |  |  |  |  |  |  |  |  |  |  |  |  |  |  |
|  | Kiss Me |  |  |  |  |  |  |  |  |  |  |  |  |  |  |  |
| Ceremonial | Kiss Me |  |  |  |  |  |  |  |  |  |  |  |  |  |  |  |
|  | Kenkō |  |  |  |  |  |  |  |  |  |  |  |  |  |  |  |
|  | Akira |  |  |  |  |  |  |  |  |  |  |  |  |  |  |  |
|  | Jade Leaf |  |  |  |  |  |  |  |  |  |  |  |  |  |  |  |
|  | Matcha Organics |  |  |  |  |  |  |  |  |  |  |  |  |  |  |  |

**Figure S4.** Statistical differences in average epigallocatechin gallate (EGCG) concentrations among green tea extracts. White denotes no statistical differences between samples while blue (0.05 > *p* < 0.01), green (0.01 > *p* <0.001), and orange (*p* < 0.001) indicate different levels of statistical significance.

|  |  | Green tea | | | | | Culinary | | | | | Ceremonial | | | | |
| --- | --- | --- | --- | --- | --- | --- | --- | --- | --- | --- | --- | --- | --- | --- | --- | --- |
|  |  | Allegro | 365 | Twinings | Pure Leaf | Lipton | Zen Spirit | Kenkō | Matcha Wellness | Jade Leaf | Kiss Me | Kiss Me | Kenkō | Akira | Jade Leaf | Matcha Organics |
| Green tea | Allegro |  |  |  |  |  |  |  |  |  |  |  |  |  |  |  |
|  | 365 |  |  |  |  |  |  |  |  |  |  |  |  |  |  |  |
|  | Twinings |  |  |  |  |  |  |  |  |  |  |  |  |  |  |  |
|  | Pure Leaf |  |  |  |  |  |  |  |  |  |  |  |  |  |  |  |
|  | Lipton |  |  |  |  |  |  |  |  |  |  |  |  |  |  |  |
| Culinary | Zen Spirit |  |  |  |  |  |  |  |  |  |  |  |  |  |  |  |
|  | Kenkō |  |  |  |  |  |  |  |  |  |  |  |  |  |  |  |
|  | Matcha Wellness |  |  |  |  |  |  |  |  |  |  |  |  |  |  |  |
|  | Jade Leaf |  |  |  |  |  |  |  |  |  |  |  |  |  |  |  |
|  | Kiss Me |  |  |  |  |  |  |  |  |  |  |  |  |  |  |  |
| Ceremonial | Kiss Me |  |  |  |  |  |  |  |  |  |  |  |  |  |  |  |
|  | Kenkō |  |  |  |  |  |  |  |  |  |  |  |  |  |  |  |
|  | Akira |  |  |  |  |  |  |  |  |  |  |  |  |  |  |  |
|  | Jade Leaf |  |  |  |  |  |  |  |  |  |  |  |  |  |  |  |
|  | Matcha Organics |  |  |  |  |  |  |  |  |  |  |  |  |  |  |  |

**Figure S5.** Statistical differences in average epigallocatechin (EGC) concentrations among green tea extracts. White denotes no statistical differences between samples while blue (0.05 > *p* < 0.01), green (0.01 > *p* <0.001), and orange (*p* < 0.001) indicate different levels of statistical significance.

|  |  | Green tea | | | | | Culinary | | | | | Ceremonial | | | | |
| --- | --- | --- | --- | --- | --- | --- | --- | --- | --- | --- | --- | --- | --- | --- | --- | --- |
|  |  | Allegro | 365 | Twinings | Pure Leaf | Lipton | Zen Spirit | Kenkō | Matcha Wellness | Jade Leaf | Kiss Me | Kiss Me | Kenkō | Akira | Jade Leaf | Matcha Organics |
| Green tea | Allegro |  |  |  |  |  |  |  |  |  |  |  |  |  |  |  |
|  | 365 |  |  |  |  |  |  |  |  |  |  |  |  |  |  |  |
|  | Twinings |  |  |  |  |  |  |  |  |  |  |  |  |  |  |  |
|  | Pure Leaf |  |  |  |  |  |  |  |  |  |  |  |  |  |  |  |
|  | Lipton |  |  |  |  |  |  |  |  |  |  |  |  |  |  |  |
| Culinary | Zen Spirit |  |  |  |  |  |  |  |  |  |  |  |  |  |  |  |
|  | Kenkō |  |  |  |  |  |  |  |  |  |  |  |  |  |  |  |
|  | Matcha Wellness |  |  |  |  |  |  |  |  |  |  |  |  |  |  |  |
|  | Jade Leaf |  |  |  |  |  |  |  |  |  |  |  |  |  |  |  |
|  | Kiss Me |  |  |  |  |  |  |  |  |  |  |  |  |  |  |  |
| Ceremonial | Kiss Me |  |  |  |  |  |  |  |  |  |  |  |  |  |  |  |
|  | Kenkō |  |  |  |  |  |  |  |  |  |  |  |  |  |  |  |
|  | Akira |  |  |  |  |  |  |  |  |  |  |  |  |  |  |  |
|  | Jade Leaf |  |  |  |  |  |  |  |  |  |  |  |  |  |  |  |
|  | Matcha Organics |  |  |  |  |  |  |  |  |  |  |  |  |  |  |  |

**Figure S6.** Statistical differences in average epicatechin concentrations among green tea

extracts. White denotes no statistical differences between samples while blue (0.05 > *p* < 0.01), green (0.01 > *p* <0.001), and orange (*p* < 0.001) indicate different levels of statistical significance.

|  |  | Green tea | | | | | Culinary | | | | | Ceremonial | | | | |
| --- | --- | --- | --- | --- | --- | --- | --- | --- | --- | --- | --- | --- | --- | --- | --- | --- |
|  |  | Allegro | 365 | Twinings | Pure Leaf | Lipton | Zen Spirit | Kenkō | Matcha Wellness | Jade Leaf | Kiss Me | Kiss Me | Kenkō | Akira | Jade Leaf | Matcha Organics |
| Green tea | Allegro |  |  |  |  |  |  |  |  |  |  |  |  |  |  |  |
|  | 365 |  |  |  |  |  |  |  |  |  |  |  |  |  |  |  |
|  | Twinings |  |  |  |  |  |  |  |  |  |  |  |  |  |  |  |
|  | Pure Leaf |  |  |  |  |  |  |  |  |  |  |  |  |  |  |  |
|  | Lipton |  |  |  |  |  |  |  |  |  |  |  |  |  |  |  |
| Culinary | Zen Spirit |  |  |  |  |  |  |  |  |  |  |  |  |  |  |  |
|  | Kenkō |  |  |  |  |  |  |  |  |  |  |  |  |  |  |  |
|  | Matcha Wellness |  |  |  |  |  |  |  |  |  |  |  |  |  |  |  |
|  | Jade Leaf |  |  |  |  |  |  |  |  |  |  |  |  |  |  |  |
|  | Kiss Me |  |  |  |  |  |  |  |  |  |  |  |  |  |  |  |
| Ceremonial | Kiss Me |  |  |  |  |  |  |  |  |  |  |  |  |  |  |  |
|  | Kenkō |  |  |  |  |  |  |  |  |  |  |  |  |  |  |  |
|  | Akira |  |  |  |  |  |  |  |  |  |  |  |  |  |  |  |
|  | Jade Leaf |  |  |  |  |  |  |  |  |  |  |  |  |  |  |  |
|  | Matcha Organics |  |  |  |  |  |  |  |  |  |  |  |  |  |  |  |

**Figure S7.** Statistical differences in average gallocatechin gallate (GCG) concentrations among green tea extracts. White denotes no statistical differences between samples while blue (0.05 > *p* < 0.01), green (0.01 > *p* <0.001), and orange (*p* < 0.001) indicate different levels of statistical significance.

|  |  | Green tea | | | | | Culinary | | | | | Ceremonial | | | | |
| --- | --- | --- | --- | --- | --- | --- | --- | --- | --- | --- | --- | --- | --- | --- | --- | --- |
|  |  | Allegro | 365 | Twinings | Pure Leaf | Lipton | Zen Spirit | Kenkō | Matcha Wellness | Jade Leaf | Kiss Me | Kiss Me | Kenkō | Akira | Jade Leaf | Matcha Organics |
| Green tea | Allegro |  |  |  |  |  |  |  |  |  |  |  |  |  |  |  |
|  | 365 |  |  |  |  |  |  |  |  |  |  |  |  |  |  |  |
|  | Twinings |  |  |  |  |  |  |  |  |  |  |  |  |  |  |  |
|  | Pure Leaf |  |  |  |  |  |  |  |  |  |  |  |  |  |  |  |
|  | Lipton |  |  |  |  |  |  |  |  |  |  |  |  |  |  |  |
| Culinary | Zen Spirit |  |  |  |  |  |  |  |  |  |  |  |  |  |  |  |
|  | Kenkō |  |  |  |  |  |  |  |  |  |  |  |  |  |  |  |
|  | Matcha Wellness |  |  |  |  |  |  |  |  |  |  |  |  |  |  |  |
|  | Jade Leaf |  |  |  |  |  |  |  |  |  |  |  |  |  |  |  |
|  | Kiss Me |  |  |  |  |  |  |  |  |  |  |  |  |  |  |  |
| Ceremonial | Kiss Me |  |  |  |  |  |  |  |  |  |  |  |  |  |  |  |
|  | Kenkō |  |  |  |  |  |  |  |  |  |  |  |  |  |  |  |
|  | Akira |  |  |  |  |  |  |  |  |  |  |  |  |  |  |  |
|  | Jade Leaf |  |  |  |  |  |  |  |  |  |  |  |  |  |  |  |
|  | Matcha Organics |  |  |  |  |  |  |  |  |  |  |  |  |  |  |  |

**Figure S8.** Statistical differences in average epi/catechin gallate (E-CG) concentrations among green tea extracts. White denotes no statistical differences between samples while blue (0.05 > *p* < 0.01), green (0.01 > *p* <0.001), and orange (*p* < 0.001) indicate different levels of statistical significance.
